# The usefulness of traditional birth attendants to women living with HIV in resource-poor settings: the case of Mfuwe, Zambia

**DOI:** 10.1186/s41182-017-0076-3

**Published:** 2017-11-13

**Authors:** Choolwe Muzyamba, Wim Groot, Sonila M. Tomini, Milena Pavlova

**Affiliations:** 1 Maastricht Graduate School of Governance/UNU-Merit, Maastricht University, Maastricht, the Netherlands; 2 A9 Marshlands Village, P.O. Box 32379, Lusaka, Zambia; 3 Department of Health Services Research, CAPHRI, Maastricht University Medical Center, Faculty of Health, Medicine and Life Sciences, Maastricht University, Maastricht, the Netherlands; 4 Top Institute for Evidence-Based Education Research (TIER), Maastricht University, Maastricht, the Netherlands; 5 Department of Economics, University of Liege, Liege, Belgium

**Keywords:** Maternal health, Traditional birth attendants (TBAs), HIV/aids

## Abstract

**Background:**

Although there is increased attention on the role of trained traditional birth attendants (TBAs) in maternal care, most of the research has mainly focused on providing evidence of the relevance of trained TBAs to women in general without a specific focus on women who are HIV positive, despite them being most vulnerable. Therefore, the aim of this study is to fill this gap by assessing the relevance of trained TBAs to women living with HIV in resource-poor settings by using Zambia as a case study.

**Methods:**

Our data collection consisted of two focus group discussions, one involving HIV-positive women utilizing trained TBAs and the other with women not utilizing TBAs. Additionally, in-depth interviews were conducted with trained TBAs and health workers. Thematic analysis was used to analyze the data.

**Results:**

In general, women living with HIV positively characterized the services of TBAs. In the face of an inefficient health system, trained TBAs were seen to be useful in providing efficient, cheap and quality care, counseling, and referral and logistical support, including treatment adherence support.

**Conclusion:**

In Zambia, trained TBAs and professional care are not mutually exclusive but complementary. There is no doubt that HIV-positive women need professionals to handle complications and offer antiretroviral treatment to ensure prevention of mother to child transmission (PMTCT). However, additional “soft” services offered by trained TBAs are equally important in the promotion of maternal health care among HIV-positive women. Thus, it seems there is more to gain by systematically allowing trained TBAs to work alongside professionals in a well-coordinated and complementary manner.

## Background

In sub-Saharan Africa, questions of who should assist during childbirth as well as where and under what circumstances childbirth should take place have long been debated [1]. Central to this debate is the role that trained traditional birth attendants (TBAs) should and could play during this process. The World Health Organization (WHO) defines trained TBAs as individuals within the community who assist mothers in maternal care and have learned their skills through apprenticeship which mostly involves informal training on how to take care of pregnant women, conduct deliveries, and provide postnatal services [2, 3]. Trained TBAs are distinguished from other TBAs who never received any training.

Scholars remain divided on the usefulness of trained TBAs especially for women with HIV who have extra needs (antiretroviral treatment to ensure prevention of mother to child transmission—PMTCT). On one hand, there is a strand of scholars [4] who argue that trained TBAs lack the necessary knowledge and skills to promote maternal health in women with HIV. Consequently, this narrative by TBA opponents has dominated the maternal health discourse in countries like Zambia as epitomized by the transposition of this policy into national maternal health policy (which emphasizes institutional delivery) [5, 6].

On the other hand, another strand of scholars [7, 8] believes that trained TBAs are indispensable in resource-poor settings like Zambia, especially considering the fact that in such areas, health facilities are remotely located, poorly staffed, poorly equipped, overcrowded, and inaccessible. This makes trained TBAs the only feasible, practical, and accessible source of maternal health care. Some scholars emphasize this point by suggesting that any attempt to abandon trained TBAs in resource-poor settings creates a danger of wiping out the only available source of care [7–9]. However, questions on whether trained TBAs can be relevant to women living with HIV in resource-poor settings remain unanswered. Therefore, the aim of this study is to help fill this gap by assessing the relevance of trained TBAs to women living with HIV in resource-poor settings by using Mfuwe, Zambia as a case study.

## Methods

We locate our assessment with the help of the framework of public value in health care provision [10]. The public value framework to health care emphasizes that the relevance of a health care service is seen from its localized interpretation and context-specific responsiveness to local needs [11]. The framework broadly allows local people to characterize a service as relevant or not by relying on their own interpretation of its value given their local needs. The localized interpretation is usually assessed in terms of quality, access, efficiency, and costs of the service. We thus use the public value framework as a heuristics device guiding our analysis and presentation of results.

### Ethical clearance

We obtained ethical clearance from the National Health Research Authority of Zambia and from the Zambian ERES Converge IRB. Other than that, informed consent was sought from the participants before participation. Further, participants were informed of their right to discontinue their participation at any point should they wish to do so.

### Study site

Mfuwe is one of the rural settlements situated in the South Luangwa national park in the Eastern province of Zambia. The main health center in Mfuwe is called “Kamoto Hospital”. This hospital caters for a population of over 207,000 people spread roughly over an area of 370 km^2^ [12, 13]. According to the 2013–2014 Zambia Demographic Health Survey (ZDHS), it is estimated that over 70% of the people in Mfuwe live in abject poverty. The HIV prevalence rate in this area ranges from 10.6 to 14%, whereas the maternal mortality rate (MMR) stands at 398 deaths per 100,000 live births [13].

### Sampling

In recruiting our participants, we relied on convenient and purposive sampling techniques. In total, we recruited 40 participants. In order to increase the diversity of our responses, participants were divided into four groups: (a) HIV-positive women who used trained TBAs (*n* = 12), (b) HIV-positive women who never used trained TBAs (*n* = 11), (c) trained TBAs (*n* = 8), and (d) health workers (*n* = 9). In general, our participants varied in age, educational level, marital status, and occupation. We ensured variety in participants in order to increase the diversity of opinions expressed.

### Interviews and focus group discussions

Focus group discussions (FGDs) were conducted separately with groups A (TBA utilizers) and B (non-TBA utilizers). Further, we conducted individual interviews separately with groups C (trained TBAs) and group D (health workers).

FGDs lasted for an average of 90 min while interviews lasted for 40 min. All interviews and FGDs were conducted by the first author, a Zambian national, in Chichewa (the local language) and English where possible. A topic guide was used during the FGDs and interviews. The guide included about 11 broad questions ranging from reasons why participants chose to use or not use trained TBAs, women’s experiences with the given service (TBAs or professionals) during pregnancy, childbirth, and postpartum. Other questions centered on women’s thoughts regarding benefits and disadvantages of the type of service used etc. The topic guide used to interview trained TBAs and professional health workers broadly consisted of questions asking them to describe what services they offered, reasons why those services were offered, experiences encountered during service provision etc. During both FGDs and interviews, we ensured that follow-up questions were asked for clarification and also to encourage respondents to expand upon their answers. The participants in the FGDs came from different ethnic groups but they all spoke the common language of Chichewa and basic English. Interviews were conducted in Chichewa, and where possible, English was used. Participants were also encouraged to express themselves freely in their ethnic dialect. We also made sure that any ethnic words and phrases used that had no English translation or good English equivalent were left un-translated.

### Analysis

We used NVivo as a software tool to conduct a thematic analysis. Thematic analysis was chosen because it effectively allows the researcher to neatly pinpoint, examine, and describe phenomenon by using themes which systematically elucidate the content of and give meaning to the data [14]. Resulting codes were progressively clustered in 34 number of organizing themes and 17 global themes (see column 1–3 in Table 2).

## Results

Table 1 summarizes key baseline demographic features of our participants. As can be seen, our participants varied in social demographic characteristics. For example, the age ranged from 18 to 63 years with the majority of them being in their twenties and thirties. In terms of educational attainment, the range was from “no education at all” to “university graduates.” A substantial number of them had acquired primary education. The number of married and unmarried women was comparable. Similarly, the number of women in employment was somewhat comparable to that of the unemployed.

**Table 1:** Participants’ demographics

ID	Age	Education level	Employment status	Marital status
Women who had accessed services of trained TBAs during their maternity				
1	25	Secondary education	Employed	Widowed
2	23	Primary education	Unemployed	Married
3	41	No education	Employed	Divorced
4	33	No education	unemployed	Married
5	19	Vocational education	Employed	Married
6	33	Primary education	Employed	Widowed
7	26	No education	Employed	Not married
8	20	Primary education	Unemployed	Not married
9	22	Secondary education	Employed	Married
10	26	Primary education	Unemployed	Married
11	40	No education	Unemployed	Married
12	38	Secondary education	Unemployed	Married
Women who had never accessed services of trained TBAs during their maternity				
13	27	Secondary education	Employed	Married
14	24	Vocational training	Unemployed	Married
15	18	Secondary education	Unemployed	Married
16	34	Primary education	Unemployed	Married
17	40	No education	Employed	Widowed
18	30	Vocational training	Unemployed	Married
19	27	No education	Unemployed	Not married
20	23	Primary education	Employed	Not married
21	22	Secondary education	Employed	Widowed
22	29	No education	Unemployed	Not married
23	33	Secondary education	Employed	Married
	AGE	Education level	Years of practice	
Trained traditional birth attendant				
24	46	Vocational training	22	
25	56	Vocational training	19	
26	48	Vocational training	18	
27	39	Vocational training	11	
28	57	Vocational training	26	
29	53	Vocational training	20	
30	63	Secondary education	30	
31	55	Vocational training	27	
32	36	Vocational training	4	
Medical professionals				
33	48	University education	20	
34	60	University education	33	
35	57	University education	30	
36	46	University education	17	
37	53	University education	24	
38	33	University education	7	
39	26	University education	3	
40	28	University education	4	

Table 2 summarizes key findings from our study. A thorough explanation of these findings is given below.

**Table 2:** Summary of qualitative results

Respondent group	Global theme	Organizing theme	Basic findings from the FGDs and interviews
Characterization of TBAs by HIV+ women who had accessed trained TBAs	Quality of care	Quality of care	•TBAs provide personalized care
			•TBAs provide continuous care from antenatal, labor, and postnatal •TBAs help in promoting adherence to treatment •Provided supportive logistic and services
		Limitations in quality	•Lack of skills to handle complications •Lack of medical supplies and equipment to prevent mother to child transmission
	Efficiency	Efficiency	•Provided psychological, emotional, and economic support •Referred and facilitated transportation to facilities •TBAs work to end stigma, discrimination, and patriarchy through advocacy Using advocacy to challenge structural drivers to poor maternal health outcomes e.g., stigma, discrimination, and patriarchy
		Limitations in efficiency	•Lack of advanced medical equipment and supplies
	Affordability	Affordability	•Were cheaper and in some cases free •Payment or token of appreciation was not mandatory
		Limitations in affordability	•N/A
	Access	Access	•Easily accessible •Lives within the community •Always available to provide care from antenatal to postnatal
		Limitations to access	•N/A
Characterization of TBAs by HIV+ women who had never accessed trained TBAs	Quality of care	Quality of care	•Less verbally and physically abusive than professionals in facilities •Provision of personalized care
			•More guaranteed support from antenatal until postpartum •Do provide resources and food •More friendly and caring than facility-based care
		Limitations in quality	•Professionals have more knowledge and skills to handle complications than trained TBAs •Professionals have access to equipment and medical supplies that can deal with HIV vulnerability during pregnancy but TBAs do not
	Efficiency	Efficiency	•N/A
		Limitations in efficiency	•TBAs cannot help in PMTCT •TBAs cannot help easily conduct HIV tests •TBAs cannot help in the provision of ARVs •TBAs cannot help in conducting cesarean births
	Affordability	Affordability	•TBAs are cheaper than institutions where they require women to pay clinical fees •TBAs are cheaper as they do not require the patient to buy her own bucket, delivery bags
		Limitations in affordability	•N/A
	Access	Access	•Always available when called upon •Follow the patients to their home
		Limitations to access	•N/A
Characterization of trained TBAs by trained TBAs	Quality of care	Quality of care	•Provide pragmatic services in the form of psychological and emotional support •Provide adherence-to-treatment support •Provide useful maternal health information •Help in providing priority attention to HIV-positive women upon recommendation at the facility
		Limitations in quality	•Limitation in skills •Lack of access to medical supplies and equipment
	Efficiency	Efficiency	•Protection from domestic violence and abuse •Provide useful information regarding maternal health promotion and nutrition •Provide transportation where possible to facilities •Help in providing priority attention to HIV-positive women upon recommendation at the facility
		Limitations in efficiency	•N/A
	Affordability	Affordability	•Cheap •No fees required
		Limitations in affordability	•N/A
	Access	Access	•Readily available to communities •Provide services to everyone including those who would have otherwise been left unattended to
			•They view their work as a civic duty to the community
		Limitations to access	•N/A
	Policy	Provide referral services	•Provide transportation where possible to facilities •Help in providing priority attention to HIV-positive women upon recommendation at the facility
		Advantages of government policy on TBAs	•N/A
		Limitations as a result of government policy on TBAs	•Lack of support from government due to promotion of facility-based policy creates cooperation problems with professionals •Obscures training opportunities for TBAs •In conflict with government policy
Professional’s characterization of trained TBAs	Quality of care	Quality of care	•Can provide soft services, e.g., psychological and emotional support including treatment adherence support •Provision of referrals services
		Limitations in quality	•As opposed to TBAs, professionals can conduct completed procedures such as cesarean births •As opposed to TBAs, professionals can effectively handle complications •Have access to life-serving medical equipment used to conduct complicated operations
	Affordability	Affordability	More affordable than institutions
		Limitations in affordability	•N/A
	Efficiency	Efficiency	•N/A
		Limitations in efficiency	Inadequate funding to cooperation between TBAs and professionals
	Policy	Advantages of government policy on TBAs	•N/A
		Limitations as a result of government policy on TBAs	•Policy frustrates cooperation between professionals and TBAs •Counterproductive government policy on TBAs •Creates barriers in accessing the most vulnerable women in remote areas

### Women who accessed services of trained TBAs

Although the experience with trained TBAs varied among these women, we observed no major differences in opinions on the basis of age, marital status, and education level. In general, the majority of the women in this group described several benefits of using trained TBAs over facilities, and in the process also noted the shortcomings of using trained TBAs.

In summing up benefits of using trained TBAs, these women pointed out that trained TBAs were easily accessible, cheaper than institutional care, provided efficient and quality care, provided support which included referrals and transportation to facilities, and treatment adherence support.

TBAs were also praised for effectively confronting barriers (such as stigma and discrimination) to better health outcomes in women with HIV. These women argued that this particular action from trained TBAs was especially important because their HIV status made them susceptible to stigma and discrimination in their communities. Almost half of the participants from this group intimated that trained TBAs were involved in advocacy work to challenge stigma, discrimination, and patriarchy. In the words of one of the participants:

“There is still this stigma that is very bad and can cause so much pain. But you see, TBAs have been going round the villages raising awareness about HIV and how stigma and discrimination is not good especially for us HIV positive pregnant women”

Participant 7

Despite the largely positive characterization of their experiences with trained TBAs, some participants were cognizant of the fact that some complicated functions were not the preserve of trained TBAs, but rather of highly skilled medical practitioners. A clear demarcation of services between those of TBAs and those of highly skilled medical practitioners was visible in their responses:

“People like us (who are HIV positive) always need to constantly take medication (antiretroviral therapy) and have cesarean births and for such, I think doctors are more helpful”

Participant 2

### Women who have never accessed services of trained TBAs

Although HIV-positive women who utilized skilled attendants also had varied responses, in general, most of them spoke highly of professional care. Considering the vulnerability that comes with being HIV positive, TBAs were considered to be a risky option by these women. Their argument centered on the idea that TBAs were not able to give accurate advice to women with HIV regarding specific complications that may arise during pregnancy. It is for this reason that they preferred professional care over trained TBAs. In as much as they praised professional care, these women remained critical of the logistical and economic difficulties involved in accessing professional care in health facilities.

“Imagine if I experience some difficulties of whatever kind, do you think the TBA will know exactly what is wrong with me? Let alone give proper advice? Being HIV positive means that on a daily basis you need professional advice but I am not sure if TBAs can do this.”

Participant 13

“I also agree that going to facilities is really expensive and very difficult. I only managed because my partner owns a motor-bike, so imagine women without such options”

Participant 14

Although the majority of the women who gave birth in facilities praised facility-based care, some of them criticized professionals for exhibiting “bad attitude.” These women stated that they were neglected and abused by medical practitioners in health facilities. Specifically, three of them accused professionals of verbally and physically abusing them during labor.

“I was left alone without any attention when my water broke. It was in the night and the nurses were busy elsewhere. They were not present..”

Participant 16

“. ..They shouted at me and sometimes slapped me for not doing what they wanted me to do. I understand that they work under very difficult conditions too, but I think this is the job they signed up for and as such, they are required to be nicer and give very good services to their patients….”

Participant 18

### Trained traditional birth attendants

Trained TBAs stated how their functions were usually misunderstood and misrepresented by most people who usually accused them of duplicating medical professionals’ roles. Trained TBAs categorically distinguished their services from professional care by highlighting that their functions were only to provide “soft services” (such as treatment adherence support, nutritional support, counseling, psychological support, logistical support, and referrals to facilities). Their functions did not, in any, way include surgical procedures. This, therefore, meant that their services were complementally and not substitutional to those of professional care givers in health facilities.

“This is where we come in. we use the Zambulance to transport these HIV positive women to the clinics for specialized treatment. So our services are very pragmatic. We have been able to provide a range of services that include care during pregnancy, help with adherence in treatment, including provision of nutritional needs where possible.”

Participant 26.

“..We do not duplicate the work of doctors. We do not perform cesarean procedures, this is beyond us. Our services in women with HIV are straightforward”

Participant 28.

### Medical professionals

Although medical professionals, in general, were critical of trained TBAs by suggesting that services of TBAs had some shortcomings (e.g., lacked adequate knowledge), some of them acknowledged the importance of TBAs in maternal care of women with HIV. Specifically, they highlighted how useful their functions (psychological, social, and logistical support) were especially in the face of inadequate health workers.

“..TBAs are the immediate help available to women here in Mfuwe. So in most cases they are the only readily available source of help. I think there should be a way Government taps into this and officially involve them in the process of maternal care. They do provide a lot of help to HIV positive women. Most of the women who come here to the clinic are usually transported by the Zambulance organized by local TBAs. They are still a very important part of the maternal care channel. Also, when we put our patients on treatment, it’s TBAs in the villages who ensure that these patients adhere to treatment because we are not physically present....”

Participant 35.

## Discussion

In this study, we set out to assess the varied ways our respondents characterized the relevance of trained TBAs in maternal care of women living with HIV in Zambia. Our findings illustrate the fact that despite the Zambian government’s policy (which emphasizes strict utilization of professional care for women with HIV), women have continued to utilize services of trained TBAs [5, 15]. Specifically, TBAs provide the following “soft services” to women with HIV: treatment adherence support, nutritional support, counseling, psychological support, logistical support, and referrals to facilities. This means that these services form part of a pragmatic response to maternal health care in Zambia. As other scholars have pointed out [3, 16], maternal health care is a continuum which requires attention not only of medical professionals but also of TBAs (in the form of soft services) [7].

A growing body of literature [17] has consistently pointed out that most resource-poor settings can benefit from task shifting through apportioning and recognizing suitable functions of trained TBAs. In line with this, evidence from our study suggests that TBAs can help improve access to care, decongest health facilities, and counteract social barriers to antiretroviral treatment, all of which are important in promoting maternal health in women with HIV. Just as other studies have shown elsewhere [7, 9], we demonstrate that TBAs indeed do have limitations (such as lack of advanced knowledge); however, delegitimizing them because of this means that opportunities to maximize their benefits and minimize their costs are effectively lost. It thus seems clear that there is much to lose when TBAs are not recognized and regulated within the line of care, as this will effectively force them to operate without any form of regulation. It can be seen that despite government’s efforts [6] to promote exclusive institutional care, HIV-positive women have continued to rely on trained TBAs due to structural barriers to professional care in Zambia. A policy of strict institutional care for women with HIV is out of touch with the realities of Zambia.

While popular recommendations (which involve increasing access to skilled care by training more health workers and constructing more hospitals) all seem attractive and useful to HIV-positive women, the reality is that poor governments have struggled to achieve these goals. Yet, despite all these constraints, TBAs remain a feasible (albeit less regarded) option to help lessen the burden of maternal care. Other studies from Malawi [18] and Zambia [16], for example, suggest that inclusion of TBAs in the line of care has the potential to reduce maternal mortality, neonatal deaths, and stillbirths. Our study comes to the same conclusion as other studies [7, 16, 9, 19, 20] on task shifting, which is that in order to promote maternal health of women with HIV in Zambia, TBAs must officially be integrated in the existing health systems. It is clear from our study that because of the existing inefficient health system, local people characterize TBAs as useful stakeholders in improving maternal health of HIV-positive women. TBAs do not pose any threats to skilled midwives because the functions of the two in relation to maternal health of women with HIV are not mutually exclusive but complementary.

In this study, we note some potential limitations. Specifically, due to logistical and financial constraints, our findings are based only on the views of participants who were located in only one of the ten provinces of Zambia. Further, due to financial and logistical limitations again, our sample size was only composed of 40 participants. This fact may have limited the variety of experiences with trained TBAs in Zambia in general. However, we argue that our study from Zambia was adequate and relevant in giving insights into the relevance of TBAs in maternal care of HIV-positive women in rural settings.

## Conclusion

Our study shows that despite government’s efforts to promote exclusive professional care for women with HIV, women in Zambia continue to utilize trained TBAs. In Zambia, trained TBAs have consistently provided “soft services” which include treatment adherence support, nutritional support, counseling, psychological support, logistical support, and referrals to facilities. We have shown that maternal care is not a zero-sum game, but a continuum in which both trained TBAs and professionals each have complementary roles to play. In this regard, it seems profitable for the government of Zambia to systematically integrate trained TBAs in maternal care of HIV-positive women; doing this will ensure improved cooperation between different stakeholders, improved access, and quality of care. It will consequently make maternal care of HIV-positive women more efficient because integrated tasks can be allocated in a well-coordinated and regulated manner.

## Abbreviations

AIDS: Acquired immune deficiency syndrome
ART: Antiretroviral treatment
HIV: Human immunodeficiency virus
PMTCT: Prevention of mother to child transmission
SSA: Sub-Saharan Africa
TBA: Traditional birth attendants
WHO: World Health Organization
